# American Association of Obstetricians and Gynecologists

**Published:** 1905-11

**Authors:** 


**A Monthly Review of Medicine and Surgery.**

*EDITOR:*

WILLIAM WARREN POTTER, M. D.

All communications, whether of a literary or business nature, books for review and exchanges, should be addressed to the editor 284 FRANKLIN ST., BUFFALO, N. Y.

VOL. LXI. NOVEMBER, 1905. NO. 4

### American Association of Obstetricians and Gynecologists.

THE eighteenth annual meeting of this association, held at the Hotel Astor, New York, September 19, 20, and 21, 1905, proved to be a medical gathering of unusual interest. The place chosen for the meeting was everything that could be desired, and no detail that could contribute to the comfort of the members or the prosperity of the association was omitted by the management of the Hotel Astor. The college room in which the sessions were held was adapted to the needs of such a meeting, light, ventilation, good acoustics, and freedom from noise being essentials that were admirably fulfilled.

The scientific part of the meeting was pitched on a high plane as will appear by an examination of the proceeding, published in large part in the *American Journal of Obstetrics* for November, just now being sent out. It is not possible nor desirable, amongst so much excellent material, to make distinction, and especially do we not wish to speak invidiously, but we think that two of the papers will attract considerable attention. We refer to the president's address, by Dr. H. W. Longyear, in which he dealt with some new problems relating to dislocated kidney; and, to the paper by Dr. W. J. Gillette, of Toledo, relating to surgery of the liver. Other papers deserve careful reading, and all are contributory to the advancement of the branches of medical science to which they severally belong.

But our object at this time, in particular, is to speak of the annual dinner of the Association, which was served at the Hotel Astor, Wednesday evening, September 20, 1905, and which was an occasion of unusual importance. Two circumstances contributed to make it so. In the first place the presence of Surgeon-General Suzuki, of the Imperial Japanese Navy was an event of significance; and to this may be added the fact that Major Louis L. Seaman, who had been twice in the Orient during the war, gave an interesting address concerning medical military events observed in Japan and Manchuria.

Dr. Howard W. Longyear, of Detroit, president of the association, presided at the banquet, and Dr. Robert T. Morris, of New York, one of the prominent members, acted as toastmaster. Surgeon-General Suzuki, who was Chief Surgeon of Admiral Togo's fleet during the Eastern war, was introduced by the toastmaster as the representitive of a nation that had made its greatest conquests in what might be fittingly termed the humanities of war. He came as the guest of honor and on his arrivial, at 10 o'clock, the banqueters rose as one man, and received the great naval surgeon with "banzais' and waving napkins, while the musicians played a Japanese air.

Rear Admiral Suzuki apparently spoke at first with some hesitation, saying that he had spent much of his life "going up and down the sea" and was therefore not fluent with words even in his own tongue. But he had no difficulty in making his hearers understand every thought he set before them. Now and then the applause and cheers which greeted his story caused him to pause a perceptible time in sheer embarrassment.

Dr. Suzuki, in the course of his remarks, said that just previous to going into action each officer and man was required to take a bath and put on clean underwear, and to this he attributed much of the absence of wound infection in the naval service. He spoke in part as follows:

"At the beginning of the war I was with the Mikasa, the flagship of the Admiral. I knew that there was to be a war, probably. It was also apparent that it would be very serious. It was the question for me: How should the best preparation be made for the best result in treating the wounded?

"That was for me to decide. It was a great responsibility. But this is the order that I gave to the surgeons of the navy. That they should prepare to treat wounds by the aseptic method only. That they should treat wounds with sterilised water, leaving the wound alone as much as possible, washing the skin and then binding the wound with sterilised cloth—cotton cloth. There were no preparations of carbolic acid or the like. It was a great step to take—but now we are not sorry. [Applause and cheers.]

"Every vessel, from battleship to torpedo boat, had apparatus for sterilising water with steam. We followed that method all through the war, wiping off the skin with sterilised water, wrapping with the sterilised cotton, and leaving the rest to the natural healing influences. Of course if there was a bit of shell or metal in sight we would take it out—but we did not meddle much. There was not time.

"Each ship had one chief surgeon and two assistants, and several of the petty officers were trained in the work of applying the aseptic bandages. There was great difficulty in applying the work. The operations were carried on below with only electric light. There were of course proper hospital accomodations, but they were up above, where it was very dangerous to be under any conditions during a battle. The wounded came in very fast; the method had the advantages of swiftness, too. But it was like operating in a swiftly moving cab on the street; always the noise and the shock, cinders drifting across the floor and fragments of shell sometimes coming in. But all through the war we stuck by this method and we are glad.

"Ten years ago, in our war with China, we used the antiseptic method. Now, with the aseptic method, among 682 men sent to the hospital we had but thirty-two deaths. [Applause and cheers.]

"Among other observations, we found that the missile of war is itself aseptic. I have an instance. The captain of the Mikasa was wounded in the calf of the leg with a piece of shell. He said: 'I do not desire to be sent home. I stay with this ship until the end of the war.' [Laughter and applause.]

"I ordered the surgeon of the Mikasa (because to remove the fragment of shell would have required a deep and a long cut and would have delayed healing a long time) to neglect the fragment and apply the usual dressing. Now, a few days ago, a year later, he went to the hospital, an incision was made, and the fragment was removed.

"There had been no suppuration. Now, this could not have been if the fragment of shell was not aseptic. But a fragment of shell is of many irregular shapes, and so it often carries with it a bit of cloth or thread into the wound—then there is invariably suppuration. Not always was it possible to tell whether there was in the wound a piece of cloth. If suppuration started, then the surgeon said:'Ah, there is a bit of cloth,' and he would cut and remove it, and then the wound would get well.

"Gentlemen, there are many interesting things of the war, but war is not of particular interest to obstetricians and gynecologists. [Laughter.] So I have told you of only what little I have had particular experience in."

As Admiral Suzuki sat down the banqueters rose, waved their napkins, shouted "banzais", and drank to their guest and the imperial navy of Japan.

The address of Major Seaman was full of material gathered during his visits to the field over which giants strove, and of lessons drawn from a study of Japanese methods. In another place we print a paper read by Dr. Seaman at the recent meeting of the military surgeons' association, that contains the essentials of his banquet speech, and which merits careful reading.

The other speakers were Dr. L. S. McMurtry, president of the American Medical Association, Dr. Hermann J. Boldt, a prominent member of the American Gynecological Society, Dr Charles L. Reed, a former president of the American Medical Association, Dr. Brooks H. Wells, editor of the American Journal of Obstetrics, and Dr. Daniel Lewis, former State Commissioner of Health, all of whom spoke eloquently on appropriate subjects. Especially did Dr. Reed respond to an impromptu call from the toastmaster, taking his text from Dr. Seaman's speech, in a manner that thrilled his auditors with his eloquent and fervid rhetoric.

The misé en scéne was charming; the flower garden in the hollow square of the table; the music, vocal and instrumental; the decorations; and, most important of all, the ladies who occupied an improvised box during the speeches,—all these contributed to make a most enjoyable evening.

Surgeon-General Suzuki visited the Association during one of its sessions and participated in the proceedings. He was received with cheers, all uniting to do honor to the man who directed the medical department of the Japanese navy during the great eastern war.

At the conclusion of the morning session on Thursday the Association was entertained at luncheon at the Metropolitan Club by Dr. Robert T. Morris, of New York; and so ended one of the greatest meetings of the Association.

Officers for the ensuing year were elected as follows: president, John Young Brown Saint Louis; vice-presidents, James Nephew West, New York, and Frank Farrow Simpson, Pittsburg; secretary, William Warren Potter, Buffalo; treasurer, Xavier Oswald Wender, Pittsburg; members of the executive council, Robert Tuttle Morris, New York, and Howard William Longyear, Detroit.

